# Effects of astaxanthin supplementation on chemically induced tumorigenesis in Wistar rats

**DOI:** 10.1186/1751-0147-54-50

**Published:** 2012-08-30

**Authors:** Adrian F Gal, Sanda Andrei, Cristina Cernea, Marian Taulescu, Cornel Catoi

**Affiliations:** 1 Department of Pathologic Anatomy, Necropsy and Veterinary Forensic Medicine, Faculty of Veterinary Medicine, University of Agricultural Sciences and Veterinary Medicine Cluj-Napoca, Calea Mănăstur 3-5, Cluj-Napoca, 400372, Romania; 2 Department of Biochemistry, Faculty of Veterinary Medicine, University of Agricultural Sciences and Veterinary Medicine Cluj-Napoca, Calea Mănăstur 3-5, Cluj-Napoca, 400372, Romania; 3 Department of Physiology, Faculty of Veterinary Medicine, University of Agricultural Sciences and Veterinary Medicine Cluj-Napoca, Calea Mănăstur 3-5, Cluj-Napoca, 400372, Romania

**Keywords:** Carcinogenesis, Mammary tumor, Astaxanthin, N-methyl-N-nitroso-urea, Wistar rat

## Abstract

**Background:**

Astaxanthin (ASTA) is a fat-soluble xanthophyll with powerful antioxidant functions. It is extracted from e.g. salmon, an important food source for certain human populations known to have a reduced risk of tumor development. It is possible that ASTA plays a role in cancer chemoprevention in such populations. The purpose of this study was to investigate the effects of dietary ASTA on chemically induced mammary tumorigenesis using N-methyl-N-nitroso-urea (MNU) in immature Wistar rats.

**Methods:**

Thirty-six 37 days old juvenile female Wistar rats were at random allocated to 4 groups of which Groups 1 and 2 received a single dose of 55 mg MNU/kg body weight. The effects of ASTA was evaluated by giving rats of Groups 2 and 4 a dose of 50 mg ASTA/kg/day for the entire duration of the study. Group 3 rats received feed added alimentary oil.

Necropsy and histopathological examinations were carried out on each rat 14 months after the administration of MNU. Haematological values and antioxidative status were determined. Oxidative stress was evaluated by monitoring superoxide dismutase (SOD) and glutathione peroxidase (GPx) activities in hepatic tissue. Lipid peroxidation and carbonylation of proteins was determined in protein extracts from the liver.

**Results:**

Tumor development occurred only in rats of Groups 1 and 2, i.e. MNU exposed animals. Frequency of tumor development in general and average number of tumors per animal were insignificant between these two groups. Mammary gland tumors developed in equal frequencies in Group 1 and 2 rats, respectively. Although only rather few tumors were found in the mammary glands, a substantial number of other tumors were found in Group 1 and 2 rats, but at equal rates.

Biochemical analyses showed significant higher levels of GPx, malondialdehyde and dinitrophenylhydrazine in Group 1 rats that for rats in all other groups thus indicating protective effects of ASTA on MNU induced hepatic oxidative stress.

**Conclusions:**

Supplementation with ASTA did not reduce tumorigenesis induced by MNU in Wistar rats. However, supplementation with ASTA seemed to have anti-inflammatory effects.

## Background

Astaxanthin (ASTA; 3,3′-dihydroxy-β,β-carotene-4,4′-dione) is a fat-soluble xanthophyll with powerful antioxidant functions and is present in e.g. salmon, microalgae, and *Pfaffia* (yeast) [1]. Salmon, the principal dietary source of ASTA, is an important component of the traditional diets of Eskimos and certain coastal tribes in North America; these groups have shown unusually low prevalence of cancer [1]. This low cancer prevalence has been attributed to the high levels of certain fatty acids, notably eicosapentaenoic acid (EPA), originating from salmon. It is also possible that ASTA plays a role in cancer chemoprevention in these populations [1]. However, as ASTA has not typically been identified as a major carotenoid in humans, information on its possible role in tumor epidemiology is lacking. The existing data on the potential of ASTA to directly prevent cancer is limited to *in vitro* cell culture studies and a few *in vivo* rodent based studies evaluating the efficiency of ASTA in tumor development [2-4]. In studies with BALB/c mice, dietary ASTA inhibited the growth of transplanted Meth-A tumor cells in a dose-dependent fashion [4]. In a related study, Meth-A tumor cell growth was inhibited when dietary ASTA supplementation began one to three weeks prior to tumor inoculation, but not when supplementation was initiated simultaneously with tumor inoculation. These results suggest that ASTA may inhibit tumor development in the early stages but not in the later stages of progression [3].

In organic solutions, ASTA is a potent quencher of singlet oxygen, an effective inhibitor of peroxyl radical-dependent lipid peroxidation and an efficient peroxyl radical-trapping compound [5]. In addition, ASTA provides significantly more antioxidant capacity than other carotenoids [6]. ASTA has 100–500 times the antioxidant capacity of vitamin E and 10 times the antioxidant capacity of beta-carotene [7]. The involvement of oxidative and nitrosative stress is incriminated in the carcinogenesis of numerous tumor types. Reactive oxygen species and reactive nitrogen species (ROS/RNS) are involved in carcinogenesis by inducing DNA mutations and over-expression of proto-oncogenes [8]. N-Methyl-N-nitrosourea (MNU) induces locally aggressive and metastasizing mammary adenocarcinomas in a number of strains of rats [9-11] by these mechanisms and antioxidants such as ASTA are therefore expected to protect against MNU induced carcinogenesis.

Our objective was to study the action of dietary ASTA in chemically induced mammary tumorigenesis using MNU in immature Wistar rats.

## Methods

### Animals

Thirty-seven days old juvenile female Wistar rats obtained from the Cantacuzino Institute, Romania were used. The rats were kept under controlled conditions (i.e., 22–23°C, approximately 60% humidity, and 12 h light–dark cycle), had free access to a standard rodent pelleted diet (Cantacuzino Institute, Romania), tap water *ad libitum* and were allowed to acclimated to laboratory conditions before the study. The study was conducted in accordance with the European Union legislation on animal experimentation. All procedures were performed according to the practices of the Romanian Board of Animal Research, and were approved by the Committee of Animal Ethics at Faculty of Veterinary Medicine Cluj-Napoca, Romania.

### Experimental design

MNU was used to induce mammary tumors (Sigma-Aldrich Chemical Co., St. Louis, code N1517-1 G). MNU was dissolved in normal saline and used immediately. ASTA (Sigma-Aldrich Chemical Co., St. Louis, code A9335-1 G) was used as an antioxidative agent to protect against MNU associated tumor development. The lipophilic ASTA was dissolved in alimentary oil (Ulvex Sun Flower trademark) before being added to the diet.

The animals were randomly allocated to and housed in four groups (9 rats per group) and treated as follows:

Group 1 Injected with a single dose of 55 mg MNU/kg body weight (BW) intraperitonally (i.p.) and received feed without ASTA.

Group 2 Injected with a single dose of 55 mg MNU/kg BW i.p. and received feed added ASTA dissolved in alimentary oil in a dose of 50 mg ASTA/kg BW/day.

Group 3 Injected with a single dose of saline i.p. and received feed added alimentary oil.

Group 4 Injected with a single dose of saline i.p. and received feed added ASTA dissolved in alimentary oil in a dose of 50 mg ASTA/kg BW/day.

Dietary supplementation with ASTA began at the age of 33 days (i.e., 4 days earlier than MNU treatment) in order to ensure an appropriate antioxidant status. The rats of groups 2 and 4 were housed individually to ensure the consumption of the entire dose of ASTA. Rats from groups 1 and 3 were caged in groups of 9 animals.

The rats were examined once a week to evaluate the mammary glands. Animals from all four groups were exsanquinated after deep narcosis with halothane 14 months after the MNU injection due to welfare grounds (tumor progression).

### Necropsy and histopathology

Necropsy was performed and gross features of the tumors, e.g. size, consistency, location, and regional lymph nodes integrity, were recorded. Specimens of tumors and of stomach, intestine, liver, pancreas, kidney, spleen, lung, lymph nodes and central nervous system were sampled and fixed in 10% neutral buffered formalin by immersion before being processed for histology and embedded in paraffin wax. Tissue sections (5 μm) were stained by Masson’s trichrome and haematoxylin and eosin. The mammary tumors were classified according to their histopathological type and as benign or malignant [12].

### Haematology

Rats of all four groups underwent haematological evaluation by the use of an Abacus Vet Junior analyzer (Diamond Diagnostics, Holliston, MA, USA). The blood was collected from the internal angle of the eye using heparinised capillary tubes of 2–3 mm. The numbers of leukocytes and erythrocytes (cells/mm^3^), including the percentage of granulocytes and agranulocytes, were determined and compared with reference values [13].

### Biochemistry

Liver samples of all rats were collected at necropsy for biochemistry. Tissue samples were immediately placed in Petri dishes in a solution of potassium phosphate buffer, 10 mM (pH = 7.35, 37°C) and then weighed. Approximately 0.5 g hepatic tissues were homogenized (in Ultra Turrax) with a solution of 10 mM potassium phosphate buffer (pH = 7.4, 37°C) and centrifuged for 10 min at 20000 *g*. The obtained protein extracts were analyzed for total protein content, superoxide dismutase (SOD) activity, glutathione peroxidase (GPx) activity, lipid peroxidation and protein oxidation. The protein content of the extracts was determined using a Protein Assay Kit Bio-Rad, based on Bradford's method [14].

The antioxidant enzymatic activity of the extracted proteins was determined by monitoring the activity of GPx and SOD. GPx and SOD activities were achieved using the commercial kits Ransel and Ransod, respectively (Randox Laboratories Ltd., Crumlin, UK) according to the manufacturer's instructions. In both cases, a semi-automatic analyzer MasterPlus Screen (Hospitex Diagnostics, Florence, Italy) was used.

The concentration of malonyl dialdehyde (MDA), the end product of lipid peroxidation and the most frequently used marker for peroxidation, was determined in the protein extracts based on the reaction with thiobarbituric acid (TBA). Basically, in 1 ml of protein extract was added to 2 ml of TBA reagent (15% trichloroacetic acid and 0.375% thiobarbituric acid in 0.25 N HCl). Samples were maintained at 100°C for 15 min, cooled and centrifuged for 10 min at 1000 *g*. The amount of pink-colored MDA-TBA adduct was measured at 535 nm (UV–vis spectrophotometer Jenway 6315, Bibby Scientific Limited, Staffordshire, UK). The results were expressed in millimoles MDA per mg of protein and were obtained using an average absorption of 1.56x10^5^M^-1^ cm^–1/24^.

Carbonylation of the proteins was carried out by using a method based on the reaction of carbonyl groups with 2.4-dinitrophenylhydrazine (DNPH). The assays of protein carbonyls involve derivatisation of the carbonyl group with 2.4-dinitrophenylhydrazine (DNPH), which leads to the formation of a 2.4-dinitrophenylhydrazone product. In a centrifuge tube, 0.5 ml of protein extract and 0.5 ml of 10% trichloroacetic acid were measured. The obtained sample was centrifuged for 10 min at 2000 *g*. The supernatant was mixed with 1 ml of DNPH in 2 N HCl and stored for 1 h at room temperature (around 20°C). Stained compounds were assayed photometrically at λ= 370 nm, against 2 N HCl (UV–vis spectrophotometer Jenway 6315). Results were expressed in micromoles DNPH per mg of protein and were obtained using an average absorption of 21 mM^–1^ cm^–1^.

### Statistics

The data obtained was interpreted statistically using parametric one way ANOVA post test (Bonferonni Multiple Comparisons Test). A confidence level of 95% (*P* < 0.05) was considered significant. Statistical evaluation compared all groups.

## Results

### Mammary tumor development

Signs of acute toxicity were not seen in any of the animals. All animals survived to the end of the study, except for a Group 2 rat that showed severe clinical signs while been handled. A couple of hours later the rat was humanely killed and necropsy revealed congenital cardiac anomalies leading to cardiac dilation. This rat was excluded from the study.

Tumor development occurred only in rats of Groups 1 and 2, i.e. MNU exposed animals. Frequency of tumor development and average number of tumors per animal were insignificant between these two groups (*P* > 0.05) (Table 1). All 9 rats of Group 1 developed tumors compared to 6 out of 8 rats in Group 2 (*P* > 0.05). Three Group 1 rats developed just one tumor while six had multiple (average 2.33 tumors/rat) and tumor size varied from 0.2 to 7.5 cm. For Group 2, three rats had one tumor each, two rats had two tumors each, and one rat developed three tumors (average 1.25 tumors/rat). Tumor size for Group 2 rats varied from 0.1 to 4 cm (Table 1).

**Table 1:** Comparative aspects regarding tumor type and size in Wistar rats of Groups 1 (n = 9) and 2 (n = 8)

**Rat no.**	**Group 1 (exposed to MNU)**		**Group 2 (exposed to MNU and ASTA)**	
	**Tumor**	**Size (cm)**	**Tumor**	**Size (cm)**
1	- nephroblastoma	2.3	-	-
	- bronchial gland carcinoma	0.4		
	- sebaceous carcinoma	0.2		
2	- differentiated liposarcoma (large ligaments)	0.2-4	- nephroblastoma	1.6/1.2
	- liposarcoma (gastro-splenic ligament)	0.2-4		
	- liposarcoma (closely to pancreatic region)	1		
	- liposarcoma (mesentery)	1		
	- liposarcoma (inguinal lymph nodes)	1		
	- bronchial gland carcinoma	0.3		
	- nephroblastoma	1.5		
3	- hemangiosarcoma in abdominal cavity	7.5	- bronchial gland carcinoma	0.1
4	- bronchial gland carcinoma	1.5	-	-
	- nephroblastoma – right kidney	0.9		
	- hemangiosarcoma – left kidney	6.8		
5	- nephroblastoma	0.3	- nephroblastoma	0.3
	- inguinal lipoma	2.3	- differentiated liposarcoma (mediastine)	1.4/0.7
	- squamous carcinoma (inguinal region)	1.2	- differentiated liposarcoma (axillary region)	0.3
6	- simple mammary adenoma	0.5	- bronchial gland carcinoma	0.1-0.2
			- cholangiocarcinoma	0.3
7	- small cell lung carcinoma	0.3	- simple ductal papillary mammary carcinoma	0.5
			- mammary fibroadenoma	2.5/1.5
8	- simple mammary fibroadenoma	6.5	- simple ductal papillary mammary carcinoma	4
9	- simple mammary adenoma	0.3	- rat excluded from study	

Tumors were not present in rats of Groups 3 and 4 (data not included in table). MNU: N-Methyl-N-nitrosourea; ASTA: Astaxanthin.

Mammary gland tumors developed in 3/9 and 2/8 Group 1 and 2 rats, respectively (*P* > 0.05). Group 1 rats only had benign mammary tumors (2 simple adenomas and 1 mammary fibroadenoma), while two Group 2 rats had both malignant (2 simple ductal papillary mammary carcinomas) and benign tumors (1 fibroadenoma). Lobular hyperplastic mammary lesions (epitheliosis and simple adenosis) were found in 2/9 and 1/8 of Group 1 and 2 rats, respectively (*P* > 0.05).

**Figure 1:**
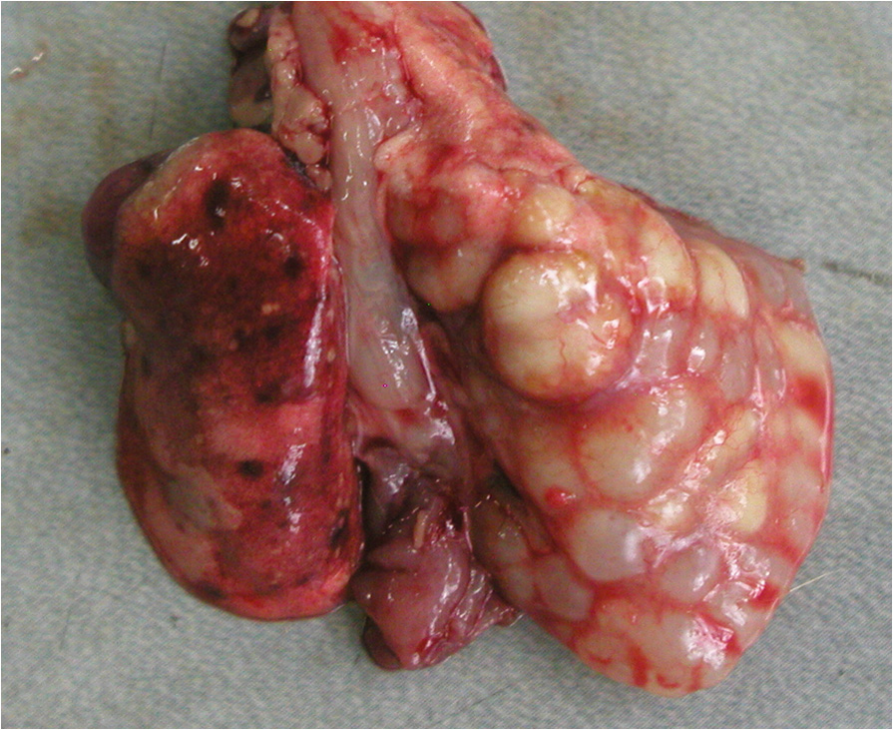
Bronchial gland carcinoma; cystic cavities containing mucus in the right diaphragmatic pulmonary lobe of a Wistar rat (Group 2; see text for explanation).

**Figure 2:**
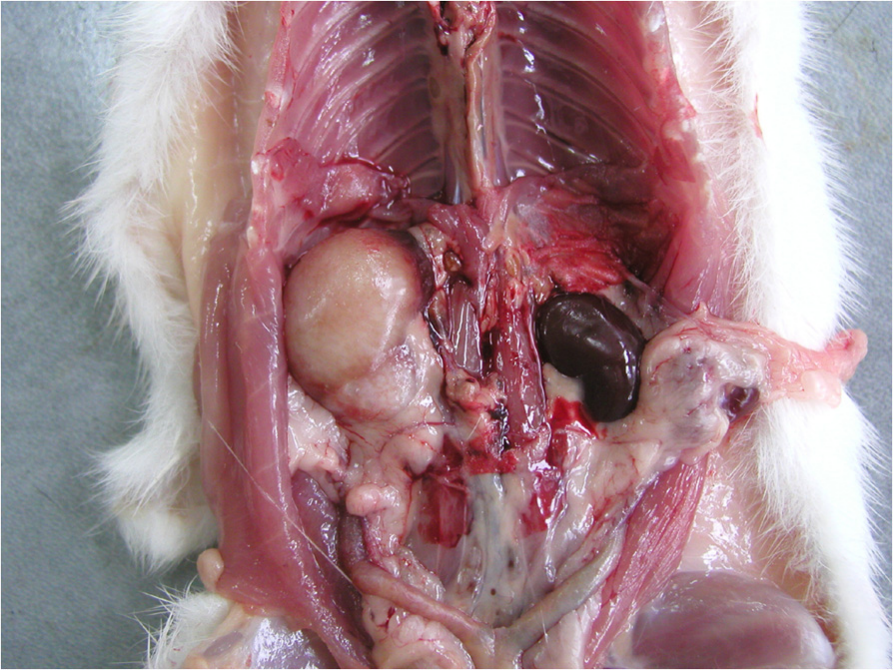
The gross aspect of a nephroblastoma of a Wistar rat (Group 2; see text for explanation) that induced hypertrophy of the right kidney.

Although only rather few tumors were found in the mammary glands, a substantial number of other tumors were found in Group 1 and 2 rats, i.e. 21 tumors in Group 1 (4 benign and 17 malignant) and 10 tumors in Group 2 (1 benign and 9 malignant). Details are provided in Table 1 and examples are shown in Figure 1 and 2.

A variety of background lesions, which are known to occur in adult Wistar rats, were found in rats of all groups in equal frequencies (*P* > 0.05) (Table 2).

**Table 2:** Background lesions in Wistar rats from the Groups 1 to 4

**Organ**	**Group 1**	**Group 2**	**Group 3**	**Group 4**
Lung	- alveolar histiocytosis	- pulmonary acinar ectasia	- alveolar histiocytosis	- alveolar hemorrhage
	- minimal multifocal mononuclear cell inflammation	- alveolar hemorrhage in the lungs	- minimal multifocal mononuclear cell inflammation	- minimal multifocal mononuclear cell inflammation
Stomach	- gastric erosions of the glandular stomach	-	- gastric erosions of the glandular stomach	-
Liver	-	- periportal lipid vacuolation	- periportal lipid vacuolation	- periportal lipid vacuolation
Pancreas	-	- acinar hyperplasia	- acinar hyperplasia	-
Central nervous system	- gliosis	- gliosis	- gliosis	- gliosis
	- cerebral hemorrhage in the vascular Virchow-Robin space			- cerebral hemorrhage in the vascular Virchow-Robin space

### Haematology

All values were within normal range according to [13]. Details are provided in Table 3. The only significant group difference was a lower number of monocytes in Group 1 *vs.* Group 3 (*P* < 0.05).

**Table 3:** Haematological data in Wistar rats from the Groups 1 to 4 (see text for details)

**Group number**		**Erythrocytes cells/mm**^**3**^	**Leukocytes cells/mm**^**3**^	**Lymphocytes%**	**Monocytes%**	**Neutrophils%**	**Eosinophils%**	**Basophils%**
Average value	1	8.102.000±1.323.924	10.724±6.912	48.8 ± 8.37	7.1 ± 2.37*A	40.1 ± 4.66	4 ± 2.81	0.3 ± 0.6
	2	8.814.285±1.593.402	13.864±3.986	57.5 ± 10	5 ± 2	31.2 ± 12	5.8 ± 3	0.3 ± 1
	3	8.615.000±631.691	9.450±1.610	61 ± 7	3.5 ± 2	26.2 ± 9	8.3 ± 2	0
	4	9.340.000±2.323.575	18.350±4.729	59.8 ± 12.12	4.7 ± 1.93	28.8 ± 13.02	6.3 ± 5.15	0.1 ± 0.25

Differences are not significant except for monocytes (Group 1 *vs.* Group 3).

A: statistic assessment of the Group 1 with the Group 3 (*P* < 0.05).

### Biochemistry

The mean values of SOD, GPx, MDA and DNPH levels are shown in Table 4. Significant higher levels were observed for SOD for Group 1 *vs.* Group 3 (*P* < 0.05) and for GPx (*P* < 0.01), MDA (*P* < 0.01) and DNPH (*P* < 0.01) for Group 1 *vs*. Groups 2, 3 and 4.

**Table 4:** Activity of antioxidative enzymes, lipidic peroxidation and proteins carbonylation in hepatic protein extracts in four groups of Wistar rats (see text for details)

**Group no.**	**Total proteins**		**Superoxidismutase (SOD)**	**Glutathion peroxidase (GPx)**	**Lipids peroxidation (MDA)**	**Proteins oxidation (DNPH)**
	**mg/ml extract**	**mg/g tissue**	**U/mg protein**	**U/mg protein**	**mmol/mg protein**	**mmol/mg protein**
1	6.787±0.40	70.911±12.61	13.453 ± 0.65*B	0.1298 ± 0.017*ABC	0.0663 ± 0.015*ABC	0.0052 ± 0.0008*ABC
2	7.474±0.45	76.895±4.79	11.225 ± 0.73	0.0348 ± 0.007	0.0449 ± 0.006	0.0032 ± 0.0005
3	8.209±0.87	83.909±9.00	10.344 ± 1.11	0.0395 ± 0.009	0.0335 ± 0.002	0.0026 ± 0.0001
4	8.105±1.20	82.165±13.45	11.270 ± 2.27	0.0538 ± 0.027	0.0302 ± 0.003	0.0022 ± 0.0004

*: *P* < 0.05 (statistically significant).

A: statistic assessment of the Group 1 with the Group 2.

B: statistic assessment of the Group 1 with the Group 3.

C: statistic assessment of the Group 1 with the Group 4.

## Discussion

Several studies indicate that MNU induces mammary tumors following i.p. exposure [9-11,15]. Our results supports this as mammary tumor prevalence in both groups treated with MNU (Groups 1 and 2) increased compared to the unexposed controls (Groups 3 and 4). Mammary tumors in this study consisted of both benign and malignant types and precancerous hyperplastic lesions were seen as well. Although others have demonstrated the utilization of MNU as a mammary tumorigenic agent in rats [16,17], there is limited data regarding extramammary tumor development. Our results show that Wistar rats may develop a wide range of tumors following MNU exposure (Table 1).

The dietary supplementation with ASTA used in this study had no significant effect in suppressing tumorigenesis induced by MNU in Wistar rats.

The biochemical results indicate that MNU exposure influenced the hepatic oxidative stress (Table 4). Overall, this was indicated by an alteration in the antioxidative systems, and increased lipidic peroxidation and proteic carbonylation for Group 1 *vs*. Groups 2, 3 and 4. In Group 2, however, dietary ASTA regulated the antioxidant enzymes (i.e., GPx, *P* < 0.01 for Group 2 *vs*. Group 1) and reduced lipid peroxidation and proteic carbonylation (i.e., *P* < 0.01 for MDA and DNPH for Group 2 *vs*. Group 1).

SOD and GPx activities were highest in the MNU treated rats of Group 1. This indicates that an antioxidant response had been activated, probably due to chronic cell changes. Such alterations could be due to a MNU induced inflammation or increased apoptosis and subsequent upregulation of these enzymes. The findings indicate that ASTA exerted an anti-inflammatory action by inhibiting ROS/RNS, although protection against MNU-induced tumorigenesis was not obtained.

## Conclusions

Supplementation with ASTA did not reduce tumorigenesis induced by MNU in Wistar rats. However, supplementation with ASTA seemed to have anti-inflammatory effects.

## Abbreviations

ASTA: Astaxanthin; DNPH: Dinitrophenylhydrazine; EPA: Eicosapentaenoic acid; GPx: Glutathione peroxidase; MDA: Malondialdehyde; MNU: N-methyl-N-nitroso-urea; ROS/RNS: Reactive oxygen species/reactive nitrogen species; SOD: Superoxide dismutase; TBA: Thiobarbituric acid test.

## Competing interests

The authors declare that they have no competing interests.

## Authors’ contributions

Before submission, all authors read and approved the final manuscript. GAF designed the study, performed all experiments, and drafted the manuscript. SA was responsible for the biochemical analyses and commented the manuscript. TM collected the materials, analyzed and interpreted the data. CrC conducted the statistical analysis. CoC revised the manuscript critically to ensure important intellectual content. GAF read and reviewed the sections and revised the manuscript. All authors read and approved the final version of the manuscript.
